# Design Principles of Inert Substrates for Exploiting Gold Clusters’ Intrinsic Catalytic Reactivity

**DOI:** 10.1038/srep15095

**Published:** 2015-10-13

**Authors:** Wang Gao, Ting Ting Cui, Yong Fu Zhu, Zi Wen, Ming Zhao, Jian Chen Li, Qing Jiang

**Affiliations:** 1 Key Laboratory of Automobile Materials, Ministry of Education, and School of Materials Science and Engineering, Jilin University, Changchun 130022, China

## Abstract

Ultralow stability of gold clusters prohibits the understanding of their intrinsic reactivity (that is vital for revealing the origin of gold’s catalytic properties). Using density functional theory including many-body dispersion method, we aim to ascertain effective ways in exploiting gold clusters’ intrinsic reactivity on carbon nanotubes (CNTs). We find that the many body van der Waals interactions are essential for gold clusters’ reactivity on CNTs and even for O_2_ activation on these supported clusters. Furthermore, curvature and dopant of CNTs are found to qualitatively change the balance between physisorption and chemisorption for gold clusters on CNTs, determining the clusters’ morphology, charge states, stability, and reactivity, which rationalize the experimental findings. Remarkably, N doped small curvature CNTs, which effectively stabilize gold clusters and retain their inherent geometric/electronic structures, can be promising candidates for exploiting gold clusters’ intrinsic reactivity.

As catalysts, gold nanoparticles have attracted great interest over past decades, due to its unexpected activity and high selectivity towards many reactions^1^,^2^,^3^,^4^. In particular, gold clusters with a few atoms Au_n_ exhibit unusual intrinsic reactivity because of their geometric and electronic structures^5^,^6^,^7^,^8^,^9^,^10^. The understanding of Au_n_ clusters’ intrinsic reactivity is of great importance, both for revealing the origin of gold’s catalytic properties and for applying gold cluster catalysts^5^,^6^,^7^. Gold clusters thus have been extensively studied in gas phase experimentally and theoretically^5^,^6^,^9^,^10^,^11^,^12^,^13^,^14^,^15^,^16^,^17^,^18^,^19^, showing that Au_n_ clusters adopt planar structures up to Au_12_^18^,^19^, while their reactivity is sensitive to size, shape, and charge states. However, the nature of catalytic properties of gold clusters is still ambiguous, since gold clusters suffer issue of ultralow stability, which leads to their short lifetimes in free states that hampers the understanding of their intrinsic reactivity practically. Many attempts have been made to stabilize metal nanoparticles by protecting them with coordinating ligands or depositing them on active substrates^20^,^21^,^22^,^23^,^24^,^25^,^26^,^27^. Although ligand-protection and active substrates support yielded some stable and active gold cluster catalysts^22^,^23^,^24^,^25^,^26^,^27^, these catalysts are entangled with the gold-ligand/gold-substrate covalent bonding, the saturation of clusters’ low-coordinate atoms, and the substantial change of clusters geometry, which blur the intrinsic reactivity of gold clusters. Therefore, it is essential to achieve the long-term stable gold clusters that retain their inherent geometric and electronic structures.

Carbon materials as inert substrates are advantageous over active substrates and have also been widely used for growing metal nanoparticles^28^,^29^,^30^,^31^. The studies by Corma *et al.* have synthesized Au_5–10_ on functionalized carbon nanotubes (CNTs) that are highly active for the aerobic oxidation of thiophenol with O_2_ but are unfortunately passivated rapidly by forming larger and inactive nanoparticles, meanwhile the Au_4_ clusters are found to be inactive on CNTs^8^. In addition, the morphology and catalytic mechanism of the supported Au_4–10_ are still unclear because of the limit of experimental detection, substantially prohibiting the attempt to improve the stability of these catalysts. Density functional approximations (DFA) with (semi-)local functionals, which miss long-range van der Waals (vdW) interactions for nonhomogeneous electron gas, cannot be applied to nanomaterials either. The main challenge for theoretically accurate determination of adsorption properties of nanomaterials originates from their nonlocal anisotropic polarization that is coupled with a pronounced contribution of many-body electronic correlations^32^. This contribution is missed in the pairwise approximation for dispersion forces too.

To reveal the properties of Au_n_ clusters on CNTs, we employ DFA augmented with accurate description of nonlocal many-body dispersion interactions (DFA+MBD) to study the configurations of Au_n_^32^,^33^, which should be affected by both chemisorption and physisorption. The possible contributions consist of covalent bonding, electrostatic interactions, Pauli repulsions, and vdW interactions. Therefore, we focus on the influence of CNTs’ curvature on Au_n_, which are accompanied by different chemical reactivity and electrodynamic response effects depending on the diameters of CNTs^34^, corresponding to distinct chemisorption and physisorption. Indeed, the curvature effect is a rather general phenomenon: Graphene often experiences curl or bending in reality due to tensions, defects, and so on^35^, intrinsically exhibiting part of CNTs.

Next, we attempt to improve the stability of Au_n_ clusters by doping CNTs (with different substitution dopants). We propose a design basis that the optimal dopant should enlarge the electrostatic attractions (but avoid forming covalent bonding) between gold clusters and substrates. Furthermore, to retain the intrinsic reactivity of gold clusters on CNTs, the candidate dopant should remain the clusters’ geometric and electronic properties unchanged compared to the isolated cases’. By investigating the influences of different dopants, we find that nitrogen dopant combined with curvature control, which perfectly meets the above requirements, can effectively accelerate the exploitation of gold clusters’ intrinsic reactivity on CNTs. In addition, our DFA + MBD calculations also provide fundamental insights into catalytic mechanism of gold clusters on CNTs.

## Results and Discussions

**Figure 1:**
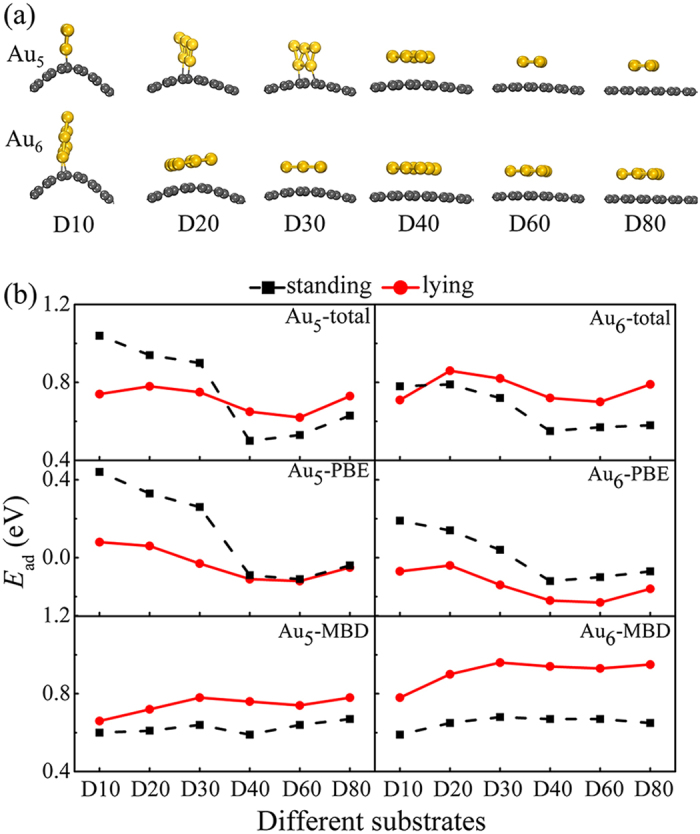
Adsorption properties of the Au_5–6_clusters on D10 ~ D80. (**a**) Configurations of the optimal Au_5–6_ clusters on D10 ~ D80. (**b**) Adsorption energies of the standing and lying modes for the Au_5–6_ clusters on D10 ~ D80. The lines guide the eye. With decreasing of the curvature (from D10 to D80), Au_5–6_ gradually transform from the standing mode to the lying mode, which are determined by the competition of chemisorption and physisorption.

We first study the influence of curvature on the morphology of the gold clusters, showing the optimal configurations and adsorption energies in Fig. 1 (more results are referred to Figure S1 and Table S1 in Supplementary Information). To simulate CNTs and curved-graphene, we use the bending graphenes, with six kinds of curvature denoted as Dm (m = 10, 20, 30, 40, 60, and 80), corresponding to the different diameters of CNTs 10 Å, 20 Å, 30 Å, 40 Å, 60 Å, and 80 Å. In addition, we choose the Au_n_ clusters with 4 ≤ n ≤ 7 that are in the range of experimental Au_3–10_^8^. To better understand the adsorption properties of Au_n_, we separate the contribution of vdW interactions (*E*_ad,MBD_) from the total adsorption energy by Perdew-Burke-Ernzerhof^36^ combined with MBD method (PBE+MBD). The rest is PBE contribution (*E*_ad,PBE_).

We find that the Au_4–7_ clusters on the substrates resemble the geometry of the isolated Au_n_ clusters, with planar structures more stable than three-dimensional ones^5^,^6^,^7^,^8^,^9^,^10^,^18^,^19^. These clusters experience two interesting adsorption modes — lying and standing — depending on the curvature of substrates (Fig. 1 and S1). We expect the further experimental studies with IR spectroscopy to identify these two modes. The Au_4_ cluster always stands on D10** **~** **D80 regardless of the curvature, while Au_5–7_ gradually transform from the standing mode to the lying mode on D10 ~ D80 with decreasing of the curvature (Fig. 1 and S1), which are determined by the competition of chemisorption and physisorption (mainly the competition of covalent bonding and vdW interactions. see Table S1). The standing mode of gold clusters forms stronger chemical bonds with substrates than the lying mode does, by optimizing the overlap of gold clusters’ the highest occupied molecular orbtials (HOMO) and substrates’ the lowest unoccupied molecular orbtials (LUMO). In contrast, the lying mode of gold clusters is conducive to maximize vdW interactions with substrates by minimizing the distance between the gold atoms and the substrates. With decreasing of the curvature, chemical reactivity of substrates gradually decreases, while vdW interactions play an increasingly important role in the adsorption of the Au_4–7_ clusters, forcing the transformation of the Au_4–7_ clusters from the standing mode to the lying mode. As the Au_n_ size increases, vdW interactions grow with 0.13 ~ 0.20 eV/atom and favor the lying mode. In particular, the lying mode Au_5–7_ experience pure physisorption on CNTs, where these clusters are anchored by vdW interactions, electrostatic interactions, and Pauli repulsions.

**Figure 2:**
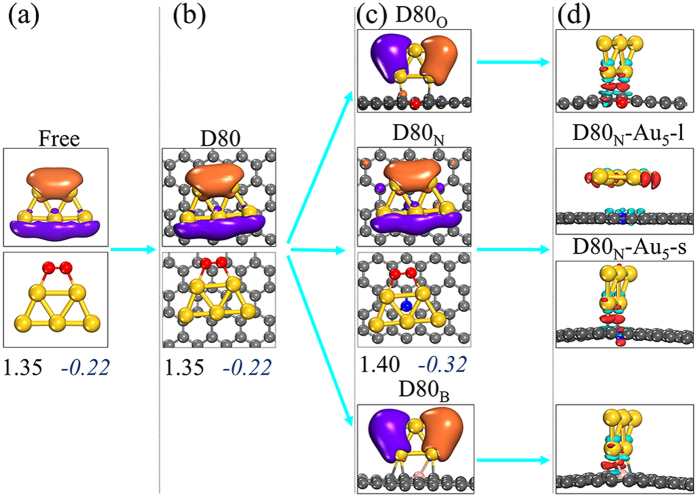
Electronic and geometric properties of the Au_5_ clusters. (**a,b**) Atomic distribution of the HOMOs of the isolated Au_5_ clusters and those supported on D80, with the optimal configurations of the adsorption of O_2_ on these clusters. (**c**) HOMOs of the Au_5_ clusters on D80_N_, D80_O_, and D80_B_ where the subscript N, O, B denote the doping element. (**d**) The electron density difference of the Au_5_ clusters on these doped D80 (both lying and standing modes for D80_N_). The different colours of HOMOs represent opposite signs of wavefunctions. LUMOs of O_2_ are not shown for simplification. For the electron density difference, blue-green (red) indicates the deletion (accumulation) of electron density. The numbers in bold are the bond lengths of adsorbed O_2_, while those in italic are the charges on O_2_ *Q* (**e**) with Hirshfeld definition.

To elucidate the reactivity of the Au_n_ clusters, we study the O_2_ activation that is crucial for plenty of reactions^7^,^8^,^9^,^10^,^12^,^13^,^14^,^15^,^16^,^17^,^37^. Our calculations demonstrate that the HOMOs of the lying mode Au_5–7_ have lobes exclusively localized on the low-coordinated Au atoms (Fig. 2 and S2), which are all accessible to the LUMO of O_2_ (LUMOs_O2_), since no covalent bonding is formed between the clusters and the substrates. As O_2_ adsorbs on these clusters, the charge states of O atoms and the lengths of O–O bond (L) are similar with those on the isolated Au_n_ clusters (Fig. 2 and S2, and Table S2), showing that the substrates greatly preserve the reactivity of the lying mode Au_5–7_. In contrast, the standing modes Au_4_ (and the standing modes Au_5_ on D10 ~ D30) bind to the substrates with covalent bonding, which saturates the low-coordinated Au atoms, making their HOMOs be partially inaccessible for LUMOs_O2_ and limiting the adsorption of other reactants (Fig. 1, S1, and S2). These results reveal the importance of taking the lying mode for the supported Au_n_ clusters to exhibit intrinsic reactivity.

On the lying modes, O_2_ activation follows the same manner as on the isolated gold clusters: O_2_ is likely activated by gold clusters with an odd (not even) number of electrons (Figure S2)^7^,^8^,^9^,^10^,^15^,^16^. In addition, the electron density, which is transferred to the π^*^_OO_ molecular orbital of O_2_, is originally from the Au_n_ clusters, while the substrates act as an electron reservoir that exchanges electrons with adsorbates during catalytic process (Tables 1 and S2). To mitigate the impact of DFT electron self-interaction error on the description of electronic properties, we adopted hybrid functional Heyd-Scuseria-Ernzerhof (HSE)^38^ to study the electron transfer among the substrates, Au_n_ clusters, and O_2_. We find that the HSE results are compatible with the PBE ones (Table 1), confirming the independence of our results on exchange-correlation functional and further supporting our conclusions. It is noteworthy that the contribution of vdW interactions to O_2_ adsorption increases from 0.06 eV on Au_4_/Dm to 0.44 eV on Au_7_/Dm with increasing of the Au_n_ size. The maximum contribution is up to 58% (0.34 ~ 0.44 eV) on Au_7_/D10 ~ D80 for the lying mode gold clusters, although it is negligible to that on the isolated gold clusters (≤0.06 eV) and the bare D10 ~ D80 (≤0.1 eV). The synergy effects of gold clusters and CNTs make vdW interactions be essential even for activating small molecules like O_2_.

The stability of the supported Au_n_ clusters is critical to the application of these clusters as catalysts, which can be evaluated from their diffusion barriers. Herein, we compute the diffusion barriers using the adsorption energy difference of the Au_n_ clusters at different adsorption sites, which are consistently less than 0.2 eV on all considered substrates (0.05 ~ 0.1 eV for the lying modes), indicating a quick migration of the supported Au_n_ clusters even at low temperature. The tiny diffusion barriers are due to the dominant role of vdW interactions in the adsorption of the Au_n_ clusters.

Overall, our results explain the experimental findings on the CNTs (the sizes correspond to D40) that the Au_5–7_ clusters are highly active for O_2_ activation but quickly aggregate into larger and inactive nanoparticles, while the Au_4_ clusters present negligible reactivity^8^. This strongly confirms the promise of our bending-graphene models in studying the properties of CNTs.

To improve the catalytic performance of the Au_n_ clusters on CNTs, we consider the role of doping in stabilizing these clusters. Elements N, O, B, P, Li, Be, Cr, and Ag are implanted into substrates respectively by replacing one C atom with one impurity atom (the concentration of 1/90). Taking Au_5_/D80 as an example, none but D80 doped with N (D80_N_) surprisingly remain the lying mode of the Au_5_ clusters (D80_N_-Au_5_-l in Fig. 2 and S2). N dopant, which is *sp*^2^ hybridization (see electron density difference of D80_N_-Au_5_-s in Fig. 2d), perfectly saturates the dangling bonds as one C atom is replaced. Therefore, the lying mode Au_5_ cluster does not form any covalent bonding with D80_N_, with the distance between D80_N_ and Au atoms around 3.36 Å. Nevertheless, the amount of electron-density 0.32e is transferred from D80_N_ to the Au_5_ cluster towards N doping (Table S2), significantly increasing electrostatic attractions (monopole-monopole and multipole-multipole interactions) between D80_N_ and Au_5_. The resulting adsorption energies for the Au_5_ cluster are *E*_ad,PBE_ = 0.19 eV and *E*_ad,MBD_ = 0.81 eV, being larger than those on D80 (*E*_ad,PBE_ = −0.05 eV and *E*_ad,MBD_ = 0.78 eV). Clearly, the N dopant stabilizes the lying mode Au_5_ on D80 by 0.27 eV without forming any covalent bonding, which remarkably meets the design basis we proposed.

In D80_N_, each N dopant contributes two *p* electrons to the *π* system and leaves the *p* orbital of N dopant unoccupied, while these two electrons are partially localized in the *p* orbital of the N dopant’s neighboring C atoms. Therefore, the standing mode Au_5_, which needs the electron-density donation from binding atoms, binds with the N dopant’s neighboring C atoms rather than the N dopant itself. In this case, electron-density is also transferred from the Au_5_ cluster to the unoccupied *p* orbital of the N dopant as shown in the D80_N_-Au_5_-s of Fig. 2d. The N dopant can hardly modify the nature of *sp*^2^ hybridization for its neighboring C atoms. Thus, the overlap of Au-5*d* and C-2*p* orbitals yields weak covalent bonding between Au_5_ and D80_N_ for the standing mode, having *E*_ad,PBE_ = 0.27 eV (*E*_ad,MBD_ = 0.64 eV) and *L*_C-Au_ = 2.36 Å. As results, the Au_5_ cluster lies on D80_N_.

In the case of the O doped D80 (D80_O_), O dopant is *sp*^3^ hybridization and has two unpaired electrons that cannot fully saturate its neighboring C atoms. Thus these C atoms undergo *sp*^3^ hybridization, leading to the overlap of Au-5*d* and C-*sp*^3^ orbitals that makes the Au_5_ cluster strongly bind with D80_O_ (*E*_ad,PBE_ = 1.32 eV). Regarding the B, Li, and Be dopants, dangling C atoms cannot be fully saturated either, which form strong covalent bonding with the Au_5_ clusters (*E*_ad,PBE_ = 1.38 ~ 2.43 eV). The sizes of P, Cr, and Ag atoms are much larger than that of C atom, making the dopants locate outside CNTs by altering bond angles of C-dopant-C rather than shortening bond lengths of C-dopant, which consequently enable forming covalent P-Au, Cr-Au, and Ag-Au bonds with *E*_ad,PBE_ of 1.14 ~ 2.20 eV.

More importantly, the optimal Au_5_ cluster on D80_N_ exhibits the identical atomic distribution of the HOMOs compared to the isolated case (Fig. 2 and S2), implying that N doped substrates retain not only the geometry but also the electronic structure of the supported clusters, which is critical for the application of gold clusters’ intrinsic reactivity. We thus adopt N as the dopant to stabilize gold clusters on CNTs.

**Figure 3:**
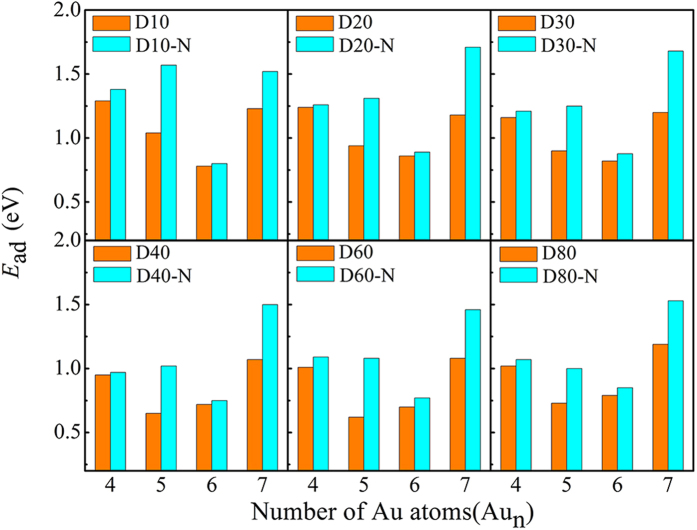
The comparison of adsorption energy for the Au_4–7_ clusters on the substrates of D10 ~ D80 and (D10 ~ D80)_N_. Adsorption energies of the Au_4–7_ clusters are overall increased towards Nitrogen doping.

As N is implanted into D10 ~ D80, the geometries of the supported Au_4–7_ are not changed. Importantly, the adsorption energies of Au_4–7_ are overall increased at doped sites (Fig. 3), especially, those of the lying modes Au_5_ and Au_7_ are increased by 0.27 ~ 0.53 eV. The resulting diffusion barriers of the Au_5_ and Au_7_ clusters are in the range of 0.35 ~ 0.63 eV on (D10 ~ D80)_N_ [2.5 ~ 10 times larger than those on (D10 ~ D80)], indicating a significantly improved stability of the Au_5_ and Au_7_ clusters even at elevated temperature of 200 ~ 350 K. The increase of adsorption energy exhibits an odd-even alteration depending on the number of gold atoms, which reflects the redistribution of electron density towards N doping. The open-shell Au_5_ and Au_7_, which can readily accept more electrons from the substrates than the close-shell Au_4_ and Au_6_ do, effectively stabilize Au_5_/(D10 ~ D80)_N_ and Au_7_/(D10 ~ D80)_N_. This stabilization can hardly change the vdW interactions between Au_4–7_ and substrates (Δ*E*_ad,MBD_ ≈ 0.05 eV in Table S1). If the N concentration is increased to 1/45, the diffusion barrier of the lying mode Au_5_ is further increased to 0.76 eV on D80_N_ (0.35 eV at concentration of 1/90).

The Au_4–7_/(D10 ~ D80)_N_ are more active in activating O_2_ compared to Au_4–7_/D10 ~ D80: more electrons (0.02 ~ 0.12 e) are transferred to O_2_ and the bond length of adsorbed O_2_ are elongated further by 0.01 ~ 0.05 Å towards N doping (Fig. 2 and S2). Namely, nitrogen dopants effectively improve the reactivity of the supported Au_n_ clusters by making the substrates donate more electrons for activating O_2_. Furthermore, our HSE calculations predict the same trend as PBE calculations for the electron transfer among O_2_, Au_5_/Au_6_, and D40_N_ (Table 1). In addition, HSE functional also identifies a comparable diffusion barrier for the Au_5_ cluster on D40_N_ as PBE functional (0.42 eV vs 0.47 eV at concentration of 1/90). Clearly, our results are independent on exchange-correlation functionals, explicitly confirming that N dopant is able to promote both reactivity and stability of the gold clusters on CNTs. In addition, the dispersed N dopant can also enhance the dispersion of the gold clusters. Recalling that the experiments of the aerobic oxidation of thiophenol with O_2_ were carried out at 300 K^8^, we also performed *ab inito* molecular dynamics simulations for Au_5_/D40_N_ and Au_7_/D40_N_ at 300 K, finding slight change of the adsorption position of the Au_5_ and Au_7_ clusters during 10 ps runs. Therefore, we conclude that the N doped CNTs largely mitigate the problem of low stability of the gold clusters on CNTs^8^ and serve as promising substrates for the application of the gold clusters’ intrinsic reactivity.

It is gratifying that CNTs and graphene exhibit sufficient flexibility towards doping functionalization^39^,^40^, while the N doped CNTs and graphene have been synthesized experimentally and exhibit remarkable reactivity for variant reactions^41^,^42^,^43^. In particular, negatively charged 10 nm gold nanoparticles were successfully anchored to the N doped CNTs^43^, while the Au_3–10_ clusters have been synthesized on CNTs^8^. These encouraging results show robust prospects for growing gold clusters on the N doped CNTs and graphene.

By comparing the MBD results to those by the pairwise approximation of Tkatchenko-Scheffler (TS)^44^ method (Table S1), we find that many body effects are essential for the lying mode Au_n_ (20 ~ 35% contribution) not for the standing mode, reflecting the highly anisotropic polarization of gold clusters and CNTs. In the case of O_2_ adsorption on these gold clusters, many body effects can critically affect O_2_ activation with up to 41% contribution on Au_7_/D80 (Table S3). In particular, these effects first reduce and then enhance the adsorption energy with decreasing of the curvature, indicating the significant influence of anisotropic polarization of nanomaterials on O_2_ activation. Recalling the dominant role of MBD force in anchoring gold clusters on CNTs and activating O_2_ on Au_n_/CNTs, DFA+MBD methods are thus essential for accurate prediction of properties of gold clusters on nanomaterials as well as catalysis on these catalysts.

## Conclusions

In conclusions, our DFA+MBD results reveal the fundamental mechanism of Au_4–7_ in activating O_2_ on CNTs, where Au_4–7_ catalytic properties are determined by the balance of chemisorption and physisorption. We find that curvature and dopant of CNTs combined with the clusters size qualitatively change this balance, determining clusters’ morphology, charge states, stability, and reactivity. Remarkably, N doped small curvature CNTs, which effectively promote gold clusters’ stability by enlarging electrostatic attractions (without forming covalent bonding), retain gold clusters’ inherent geometric and electronic structures. These results enable us to explain the experimental findings of gold clusters on CNTs^8^ and to predict N doped CNTs as promising substrates for exploiting gold clusters’ intrinsic reactivity. The methodologies we employed, including the DFA+MBD method and the principles of tuning substrates for gold clusters, can serve as a tool to engineer other clusters (*i*.*e*. Pt_n_) supported on nanomaterials where strong covalent bonding ought to be avoided.

## Methods

vdW interactions were calculated in the scheme of non-local MBD method on top of DFA (DFA+MBD)^32^,^33^, using the FHI-aims all electron code with “tight” settings^45^. The DFA+MBD approaches add the vdW energy given as a sum of *C*_6_*R*^−6^ terms to the DFA total energy. The DFA+MBD methods compute the long-range correlation energy through the coupled harmonic oscillator model Hamiltonian^32^,^33^,^46^,^47^,^48^, which is an effective random phase approximation-like treatment of many body effect, going beyond the pairwise vdW approaches. In addition, the MBD approach, which avoids the explicit use of single-electron orbitals, allows for a favorable *N*^3^ scaling (*N* is the number of atoms) and a negligible computational cost relative to a self-consistent DFA calculation. More importantly, The MBD methods has been found to be highly accurate for many molecular and solid-state systems^32^,^33^,^46^,^47^,^48^.

All geometries were obtained using CASTEP^49^ code with Vanderbilt-type ultrasoft pseudopotentials^50^ and the PBE + TS method^36^,^44^. Forces and stresses for TS calculations were calculated numerically and used to obtain fully consistent TS geometries for all calculations^44^: the normal self-consistency cycle is first completed for PBE; second, the resulting self-consistent electron density is used to create the vdW energy; After adding this vdW energy to PBE total energy, one can effectively compute the forces of PBE+TS. The PBE+TS method has been found to yield the interlayer distance of 3.34 Å for graphite^51^, in perfect agreement with the experimental value 3.34 Å. The careful convergence tests allow us to adopt a cutoff energy of 400 eV and a *k*-point mesh of 2 × 2 × 1 for 90 atoms supercell of graphene (5 × 9). All calculations are spin unrestricted. The DFA + MBD calculations are shown to converge MBD energy to a meV/atom level. To explicitly elucidate the interplay between substrates and gold clusters, we also compute the electronic structures of considered systems using hybrid HSE functional^38^.

To obtain the most stable structures of gold clusters on substrates, we defined adsorption energy of gold clusters (*E*_ad_) as:


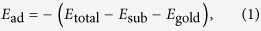


where *E*_total_ is the energy of the bound Au_n_/substrates, *E*_sub_ is the energy of the substrates, and *E*_gold_ is the energy of the isolated, fully relaxed Au_n_ clusters. Since the vdW energy is added as an additional term to the PBE total energy, it is thus convenient to separate the vdW contribution (by MBD) from the total adsorption energy.

## Additional Information

**How to cite this article**: Gao, W. *et al.* Design Principles of Inert Substrates for Exploiting Gold Clusters’ Intrinsic Catalytic Reactivity. *Sci. Rep.* **5**, 15095; doi: 10.1038/srep15095 (2015).

## Supplementary Material

Supplementary Information

## Figures and Tables

**Table 1:** Hirshfeld charges *Q* (e) of the Au_5_, Au_6_, O_2_/Au_5_, and O_2_/Au_6_ complexes on D40 and D40_N_ where the subscript N denotes the doping element, compared to the corresponding results on the isolated gold clusters.

Species		Free		D40		D40_N_	
		PBE	HSE	PBE	HSE	PBE	HSE
Au_5_	*Q*_Au5_	0	0	0.03	0.13	−0.33	−0.31
O_2_/Au_5_	*Q*_Au5_	0.22	0.40	0.35	0.38	0.19	0.30
	*Q*_O1_	−0.11	−0.20	−0.11	−0.13	−0.17	−0.16
	*Q*_O2_	−0.11	−0.20	−0.11	−0.13	−0.17	−0.16
Au_6_	*Q*_Au6_	0	0	0.17	0.16	0.05	0.14
O_2_/Au_6_	*Q*_Au6_	0.07	0.03	0.17	0.17	0.02	0.13
	*Q*_O1_	−0.03	−0.01	−0.05	−0.01	−0.10	−0.04
	*Q*_O2_	−0.04	−0.02	−0.06	−0.02	−0.12	−0.05

O1 and O2 indicate the oxygen atom of adsorbed O_2_. Both PBE and HSE results are shown for comparison purpose.
