# Supplementary material for: Design Principles of Inert Substrates for Exploiting Gold Clusters’ Intrinsic Catalytic Reactivity
Source: Sci Rep. 2015 Oct 13;5:15095. doi: 10.1038/srep15095 (PMC4602230; doi:10.1038/srep15095)
Supplement: Supplementary Information [file srep15095-s1.pdf]

Supplementary information: Design Principles of Inert Substrates for Exploiting Gold Clusters' Intrinsic Catalytic Reactivity

Wang Gao‡, Ting Ting Cui‡, Yong Fu Zhu, Zi Wen, Ming Zhao, Jian Chen Li, and Qing Jiang*

Key Laboratory of Automobile Materials, Ministry of Education, and School of Materials Science and Engineering, Jilin University, Changchun 130022, China.

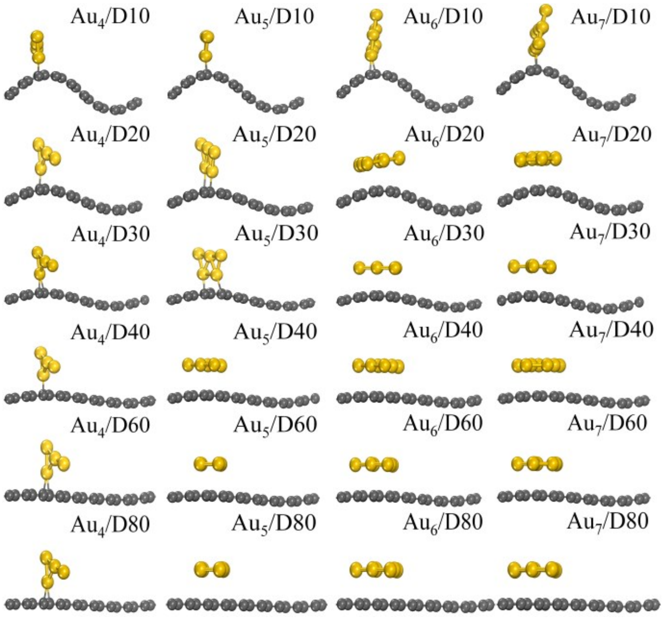

Supplementary Figure S1. Configurations of the optimal Au_{4-7} clusters on D10~D80. With decreasing of the curvature (from D10 to D80), Au_4 always stands on the substrates while Au_{5-7} gradually transform from the standing mode to the lying mode, which are determined by the competition of chemisorption and physisorption.

Supplementary Table S1. Adsorption energy of gold clusters on D10~D80. The vdW interactions contribution ($E_{ad,MBD}$) and PBE contribution ($E_{ad,PBE}$) to adsorption energies are also shown separately. –l and –s indicate the lying and standing modes respectively. $\Delta E_{ad,MBD}$ is defined as $E_{ad,MBD}$(lying) – $E_{ad,MBD}$(standing) while $\Delta E_{ad,PBE}$ is defined as $E_{ad,PBE}$(standing) – $E_{ad,PBE}$(lying).

	$E_{ad,TS}$	$E_{ad,MBD}$	$E_{ad,PBE}$	E_{ad}	$\Delta E_{ad,MBD}$	$\Delta E_{ad,PBE}$	$E_{ad,TS}$	$E_{ad,MBD}$	$E_{ad,PBE}$	E_{ad}
							Doping N			
Au_4/D10-l	--	--	--	--			0.67	0.56	0.27	0.83
Au_4/D10-s	0.54	0.52	0.77	1.29			0.57	0.54	0.84	1.38
Au_5/D10-l	0.83	0.66	0.08	0.74	0.06	0.36	0.85	0.69	0.41	1.10
Au_5/D10-s	0.56	0.60	0.44	1.04			0.54	0.58	0.99	1.57
Au_6/D10-l	0.98	0.78	-0.07	0.71	0.19	0.26	0.98	0.78	0.01	0.79
Au_6/D10-s	0.60	0.59	0.19	0.78			0.61	0.58	0.22	0.80
Au_7/D10-l	1.15	0.94	0.21	1.15	0.19	0.27	1.18	0.96	0.56	1.52
Au_7/D10-s	0.73	0.75	0.48	1.23			0.80	0.78	0.71	1.48
Au_4/D20-l	0.74	0.60	0.07	0.67	0.06	0.63	0.78	0.64	0.28	0.92
Au_4/D20-s	0.59	0.54	0.70	1.24			0.61	0.57	0.69	1.26
Au_5/D20-l	0.92	0.72	0.06	0.78	0.11	0.27	0.99	0.80	0.43	1.23
Au_5/D20-s	0.57	0.61	0.33	0.94			0.64	0.65	0.66	1.31
Au_6/D20-l	1.14	0.90	-0.04	0.86	0.25	0.18	1.12	0.88	0.01	0.89
Au_6/D20-s	0.70	0.65	0.14	0.79			0.86	0.81	0.07	0.88
Au_7/D20-l	1.37	1.17	0.01	1.18	0.41	-0.15	1.42	1.21	0.50	1.71
Au_7/D20-s	0.78	0.76	0.36	1.12			0.83	0.82	0.62	1.44
Au_4/D30-l	0.80	0.67	-0.02	0.65	0.11	0.62	0.78	0.62	0.30	0.92
Au_4/D30-s	0.61	0.56	0.60	1.16			0.63	0.58	0.63	1.21
Au_5/D30-l	1.00	0.78	-0.03	0.75	0.14	0.29	0.97	0.74	0.44	1.18
Au_5/D30-s	0.61	0.64	0.26	0.90			0.63	0.64	0.61	1.25
Au_6/D30-l	1.21	0.96	-0.14	0.82	0.28	0.18	1.18	0.93	-0.05	0.88
Au_6/D30-s	0.73	0.68	0.04	0.72			0.74	0.67	0.00	0.67
Au_7/D30-l	1.36	1.09	0.11	1.20	0.33	0.22	1.36	1.06	0.62	1.68
Au_7/D30-s	0.79	0.76	0.33	1.09			0.83	0.78	0.62	1.40
Au_4/D40-l	0.80	0.62	-0.12	0.50	0.08	0.53	0.81	0.63	0.15	0.78
Au_4/D40-s	0.60	0.54	0.41	0.95			0.63	0.56	0.41	0.97
Au_5/D40-l	1.00	0.76	-0.11	0.65	0.17	0.02	1.00	0.77	0.25	1.02
Au_5/D40-s	0.60	0.59	-0.09	0.50			0.65	0.64	0.36	1.00
Au_6/D40-l	1.22	0.94	-0.22	0.72	0.27	0.10	1.17	0.89	-0.14	0.75
Au_6/D40-s	0.75	0.67	-0.12	0.55			0.71	0.61	-0.12	0.49
Au_7/D40-l	1.36	1.05	0.02	1.07	0.28	0.12	1.38	1.06	0.44	1.50
Au_7/D40-s	0.83	0.77	0.14	0.91			0.82	0.77	0.53	1.30
Au_4/D60-l	0.82	0.64	-0.12	0.52	0.02	0.51	0.68	0.64	0.13	0.77
Au_4/D60-s	0.67	0.62	0.39	1.01			0.69	0.64	0.45	1.09
Au_5/D60-l	0.99	0.74	-0.12	0.62	0.10	0.01	1.06	0.83	0.25	1.08
Au_5/D60-s	0.64	0.64	-0.11	0.53			0.67	0.67	0.34	1.01
Au_6/D60-l	1.21	0.93	-0.23	0.70	0.26	0.13	1.28	1.09	-0.32	0.77
Au_6/D60-s	0.76	0.67	-0.10	0.57			0.77	0.67	-0.11	0.56
Au_7/D60-l	1.38	1.07	0.01	1.08	0.28	0.06	1.49	1.24	0.22	1.46
Au_7/D60-s	0.88	0.79	0.07	0.86			0.85	0.80	0.42	1.22
Au_4/D80-l	0.84	0.67	-0.08	0.59	0.05	0.48	0.84	0.66	0.14	0.80
Au_4/D80-s	0.68	0.62	0.40	1.02			0.67	0.61	0.46	1.07
Au_5/D80-l	1.02	0.78	-0.05	0.73	0.11	0.01	1.05	0.81	0.19	1.00
Au_5/D80-s	0.65	0.67	-0.04	0.63			0.63	0.64	0.27	0.91
Au_6/D80-l	1.23	0.95	-0.16	0.79	0.30	0.09	1.23	0.96	-0.11	0.85
Au_6/D80-s	0.74	0.65	-0.07	0.58			0.71	0.59	-0.08	0.51
Au_7/D80-l	1.44	1.12	0.07	1.19	0.34	0.12	1.44	1.12	0.41	1.53
Au_7/D80-s	0.83	0.78	0.19	0.97			0.82	0.77	0.25	1.02

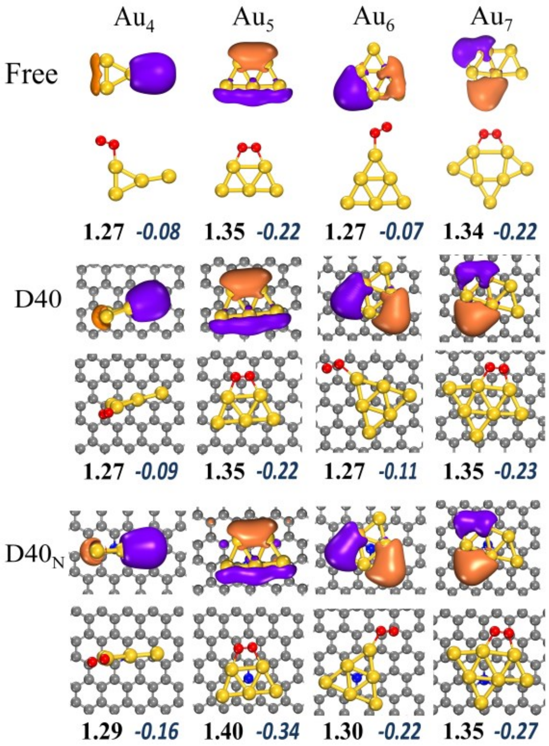

Supplementary Figure S2. Atomic distribution of the highest occupied molecular orbitals (HOMOs) of the isolated gold clusters and the gold clusters supported on D40 and $D40_N$ where the subscript N denotes the doping element, and the optimal configurations of the adsorption of O_2 on these clusters. The numbers in bold are the bond length of adsorbed O_2, while those in italic are the charge on O_2 (e) with Hirshfeld definition. The lowest unoccupied molecular orbitals (LUMOs) of O_2 are not shown for simplification. The different colours of HOMOs represent opposite signs of wavefunctions.

Supplementary Table S2. Hirshfeld charges Q (e) of the Au_n and O_2/Au_n complexes on D10~D80 and $(D10\text{-}D80)_N$ substrates, compared to the corresponding results on the isolated gold clusters. O1 and O2 indicate the oxygen atom of adsorbed O_2.

Species		Free	D10	$D10_N$	D20	$D20_N$	D30	$D30_N$	D40	$D40_N$	D60	$D60_N$	D80	$D80_N$
Au_4	Au_4	0	-0.05	-0.12	-0.05	-0.13	-0.05	-0.10	-0.05	-0.12	-0.02	-0.06	-0.02	-0.07
O_2/Au_4	Au_4	-0.08	-0.01	-0.08	0	-0.10	0.03	-0.10	-0.04	-0.11	0	-0.11	0.01	-0.10
	O1	-0.04	-0.04	-0.09	-0.04	-0.07	-0.06	-0.08	-0.07	-0.07	-0.04	-0.08	-0.04	-0.07
	O2	-0.04	-0.03	-0.07	-0.03	-0.09	-0.05	-0.09	-0.05	-0.09	-0.04	-0.09	-0.03	-0.09
Au_5	Au_5	0	0.07	-0.01	0.08	-0.05	0.08	-0.06	0.03	-0.33	0.04	-0.33	0.05	-0.32
O_2/Au_5	Au_5	0.22	0.24	0.07	0.19	0.19	0.25	0.19	0.35	0.19	0.34	0.23	0.38	0.24
	O1	-0.11	-0.12	-0.06	-0.11	-0.13	-0.12	-0.13	-0.11	-0.17	-0.11	-0.16	-0.11	-0.16
	O2	-0.11	-0.13	-0.08	-0.12	-0.14	-0.12	-0.14	-0.11	-0.17	-0.11	-0.16	-0.11	-0.16
Au_6	Au_6	0	-0.01	0.01	0.17	0.16	0.16	0.05	0.17	0.05	0.16	0.10	0.16	0.07
O_2/Au_6	Au_6	0.07	0.10	-0.01	0.19	0	0.21	0.08	0.17	0.02	0.16	0.02	0.20	0.01
	O1	-0.03	-0.05	-0.09	-0.07	-0.12	-0.06	-0.11	-0.05	-0.10	-0.07	-0.10	-0.06	-0.10
	O2	-0.04	-0.04	-0.12	-0.06	-0.10	-0.08	-0.13	-0.06	-0.12	-0.05	-0.12	-0.05	-0.12
Au_7	Au_7	0	-0.01	-0.34	0.11	-0.31	0.07	-0.34	0.08	-0.38	0.10	-0.29	0.11	-0.37
O_2/Au_7	Au_7	0.22	0.28	-0.01	0.44	0.05	0.46	0.17	0.47	0.19	0.30	0.20	0.41	0.21
	O1	-0.11	-0.10	-0.11	-0.12	-0.11	-0.12	-0.14	-0.11	-0.13	-0.15	-0.13	-0.11	-0.14
	O2	-0.11	-0.07	-0.08	-0.12	-0.13	-0.12	-0.14	-0.12	-0.14	-0.15	-0.14	-0.11	-0.14

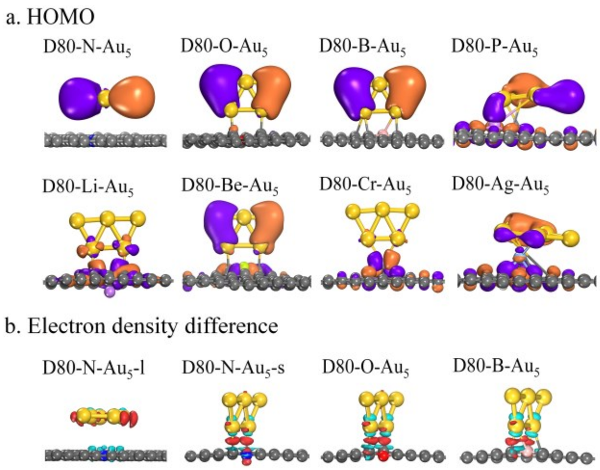

Supplementary Figure S3*.* The optimal geometry and the atomic distribution of the HOMOs of the Au_5 clusters on different doped D80 in Figure a, along with the electron density difference of the Au_5 clusters on $D80_N$ (in both lying and standing modes), $D80_O$, and $D80_B$ in Figure b. The different colours of HOMOs represent opposite signs of wavefunctions. For the electron density difference, blue-green (red) indicates the deletion (accumulation) of electron density.

Supplementary Table S3. Adsorption energies of O_2 on the Au_{4-7} clusters that are supported on D10~D80 and $(D10\sim D80)_N$, in comparison with those on the isolated gold clusters. PBE, TS, and MBD denote the results of $E_{ad,PBE}$, $E_{ad,TS}$, and $E_{ad,MBD}$.

		Au_4	Au_5	Au_6	Au_7
free	PBE	0.71	1.22	0.19	0.33
	TS	0.02	0.02	0.02	0.01
	MBD	0.03	0.06	0.03	0.05
D10	PBE	0.52	1.02	0.10	0.35
	TS	0.05	0.09	0.06	0.62
	MBD	0.08	0.08	0.10	0.44
$D10_N$	PBE	0.45	0.23	0.63	0.47
	TS	0.04	0.01	0.6	0.54
	MBD	0.07	0.03	0.23	0.24
D20	PBE	0.48	1.16	0.41	0.67
	TS	0.07	-0.05	0.18	0.26
	MBD	0.07	-0.06	0.23	0.35
$D20_N$	PBE	0.67	0.79	0.70	0.64
	TS	0.05	0.00	0.19	0.19
	MBD	0.08	-0.03	0.21	0.26
D30	PBE	0.47	1.12	0.37	0.25
	TS	0.06	0.06	0.22	0.26
	MBD	0.10	0.03	0.27	0.35
$D30_N$	PBE	0.60	0.96	0.64	0.27
	TS	0.06	0.01	0.24	0.27
	MBD	0.08	-0.02	0.28	0.36
D40	PBE	0.44	1.01	0.20	0.43
	TS	0.10	0.16	0.17	0.30
	MBD	0.14	0.24	0.2	0.40
$D40_N$	PBE	0.75	0.96	0.36	0.34
	TS	0.08	0.13	0.23	0.25
	MBD	0.1	0.14	0.27	0.35
D60	PBE	0.58	1.15	0.39	0.43
	TS	0.03	0.18	0.13	0.27
	MBD	0.04	0.19	0.12	0.36
$D60_N$	PBE	0.53	0.83	0.77	0.53
	TS	0.03	0.20	0.20	0.20
	MBD	0.05	0.28	0.28	0.24
D80	PBE	0.60	0.98	0.17	0.46
	TS	0.03	0.18	0.20	0.22
	MBD	0.04	0.19	0.28	0.34
$D80_N$	PBE	0.63	0.85	0.55	0.40
	TS	0.06	0.19	0.21	0.22
	MBD	0.08	0.23	0.26	0.33
